# Today´s medical self and the other: Challenges and evolving solutions for enhanced humanization and quality of care

**DOI:** 10.1371/journal.pone.0181514

**Published:** 2017-07-31

**Authors:** Perla Sueiras, Victoria Romano-Betech, Alejandro Vergil-Salgado, Adalberto de Hoyos, Silvia Quintana-Vargas, William Ruddick, Anaclara Castro-Santana, Sergio Islas-Andrade, Nelly F. Altamirano-Bustamante, Myriam M. Altamirano-Bustamante

**Affiliations:** 1 Grupo Transfuncional en Etica Clínica, Centro Médico Nacional Siglo XXI, IMSS, Doctores, Ciudad de México, Mexico; 2 CIECAS, Instituto Politécnico Nacional, Lauro Aguirre 120, Agricultura, Miguel Hidalgo, Ciudad de México, México; 3 Instituto de Salud Pública del Estado de Guanajuato, Tamazuca 4, Centro, Guanajuato, México; 4 Center for Bioethics, New York University, New York, NY, United States of America; 5 National Research Council for Science and Technology (CONACYT), Ciudad de México, México; 6 Instituto Nacional de Pediatría, Secretaría de Salud, Insurgentes Sur 3700, Insurgentes Cuicuilco, Ciudad de México, México; 7 Unidad de Investigación de Enfermedades Metabólicas, Centro Médico Nacional Siglo XXI, IMSS, Doctores, Ciudad de México, México; Waseda University, JAPAN

## Abstract

**Background:**

Recent scientific developments, along with growing awareness of cultural and social diversity, have led to a continuously growing range of available treatment options; however, such developments occasionally lead to an undesirable imbalance between science, technology and humanism in clinical practice. This study explores the understanding and practice of values and value clusters in real-life clinical settings, as well as their role in the humanization of medicine and its institutions. The research focuses on the values of clinical practice as a means of finding ways to enhance the pairing of Evidence-Based Medicine (EBM) with Values-based Medicine (VBM) in daily practice.

**Methods and findings:**

The views and representations of clinical practice in 15 pre-CME and 15 post-CME interviews were obtained from a random sampling of active healthcare professionals. These views were then identified and qualitatively analyzed using a three-step hermeneutical approach.

A *clinical values space* was identified in which ethical and epistemic values emerge, grow and develop within the biomedical, ethical, and socio-economic dimensions of everyday health care. Three main values—as well as the dynamic clusters and networks that they tend to form—were recognized: healthcare personnel-patient relationships, empathy, and respect. An examination of the interviews suggested that an adequate conceptualization of values leads to the formation of a wider axiological system. The role of *clinician-as-consociate* emerged as an ideal for achieving medical excellence.

**Conclusions:**

By showing the intricate clusters and networks into which values are interwoven, our analysis suggests methods for fine-tuning educational interventions so they can lead to demonstrable changes in attitudes and practices.

## Introduction

### The evidence-based medicine–Values-based medicine paradigm

Two major models have become dominant in recent years as frameworks for the practice of medicine: Evidence-Based Medicine (EBM) and Values-Based Medicine (VBM). While the former emphasizes systematic research aided by technological advancement as the basis for clinical decision making; the latter focuses on linking scientific evidence to the specific and sometimes conflicting values operating on both the patient’s side as well as the clinicians’ side during treatment [1]. To effectively fulfill the ends of medicine, i.e., healing, curing and caring [2], it is paramount that a comprehensive approach that successfully unifies the evidence-based and values-based practices is established. The need for such an approach is recognized by active healthcare professionals as well as members of the academic community. [3–5].

In the case of medical practice epistemic values are central in evidence-based medicine (in the diagnosis, treatment and prognosis of a patient) finding the cause of an illness is very important, however values-based medicine includes ethical values involved in the consequences of treatment for a patient’s life and the decisions surrounding it are also essential. In this paper, we concentrate on strengthening the pair evidence-based medicine and values-based medicine in order to get the most out of medical care.

In this paper values will be understood as normative systems that enable us to consider an object, an action, feature or a situation as something good, agreeable, convenient or desirable towards achieving certain ends [6]. Often values are thought of as exclusively ethical or only existing in relation to our dealing with others and the judging of actions as good; however, there are several kinds of values, for example aesthetical, epistemic, or economic values; all of which signal towards the given ends of these fields. Epistemic values are normative systems that enable us to characterize features or actions that one develops in the pursuit of scientific knowledge; objectivity, precision and clarity are considered valuable because they determine the kind of approach to reality a research project has, they are therefore epistemic values rather than moral ones. Putnam argues that the fact-value dichotomy is often impossible to ascertain since whenever a researcher seeks to establish a fact, values will always be intertwined with his/her research [7]. It is in this context the binomial EBM-VBM becomes an imperative to medical practice.

Regrettably, the successful integration of the EBM-VBM binomial has not yet been achieved in clinical settings around the world. Rather than being understood as complementary partners, they are often considered incompatible or even competing categories, which is why efforts must be directed at ensuring a better understanding of the interplay between epistemic and ethical values as well as their impact on the humanization of institutional medical attention. This is particularly important in times of acute vulnerability, such as situations of illness, pain, suffering, and death (IPSD).

### Bringing professionalization and ethics together

As previously noted, the emphasis currently being placed upon professionalization on the one hand and attention to medical ethics on the other, calls for an empirical investigation that is able to contribute to a more successful amalgamation of the theoretical, ethical, and practical dimensions of healthcare research [8].

Ethical training and values-awareness feature more prominently than ever in the undergraduate and graduate curricula of future clinicians [9]. However, there is still a pressing need to implement more educational interventions on active healthcare practitioners, especially among those working in public institutions and who face an ever-demanding workload on a daily basis.

The extant literature on professionalism and Continuing Medical Education (CME) generally focuses on the experiences and challenges of various sectors of clinical practice [10], but a comprehensive understanding that leads to more effectual sensitization can only be achieved by targeting the broadest array of participants as possible. This consideration lies at the heart of the design of our research, which accounts for the views, needs, and values of physicians, nurses, social workers, and other active members of the healthcare team in public sector hospitals.

Finally, we begin by accepting the premise that the ethical values of healthcare professionals stem from their life history, their professional training, and daily work [11,12]. Healthcare professionals have shared values among themselves and with their patients. It is, therefore, a matter of promoting an active recognition of the values that are already in operation within their daily practice; this recognition involves the conceptualization, appropriation, and enhancement of said values. Finding an effective way of raising awareness about dormant values and unpacking prominent values—along with their extended networks—is very important and is precisely one of the goals of this research.

In this paper, we present the findings that arose from the hermeneutic analysis of interviews before and after an educational intervention in the form of a voluntary online course in clinical ethics that was carried out in a broad array of public sector hospitals in Mexico. This systematized enquiry reveals the array of values that are already present in active clinicians in their relationships with patients and how value-awareness goes hand-in-hand with an enhanced humanization of the patient experience in condition of IPSD. This in-depth analysis of empirical material will broaden our understanding of the conceptual and practical issues of day-to-day healthcare and the key contact points of the EBM-VBM binomial, thus contributing to its solidification. Having identified and conceptualized these issues individually and as part of a network, we aim to ascertain the areas where EBM and VBM intersect and the characteristics of an ideal clinician-patient relationship that guarantees medical excellence from both a scientific and a humane perspective.

## Materials and methods

To attain an empirical understanding of the interplay between the healthcare team’s personal and work values and their professional practice, a hermeneutic approach was chosen to assess two sets of interviews concerning aspects of the life history of healthcare personnel, the healthcare professional-patient relationship, and ethical dilemmas arising around IPSD in the clinical setting. (Coreq Check list is in S1 Table)

### Design of the instrument

Upon discovering that there is no ethnographic approach to studying the values of healthcare professionals at an institutional level in the literature [13–15], we began gathering qualitative information that could be translated into measurable indicators. The instrument was designed by a trans-functional panel of experts in different areas of knowledge, the panel was pilot-tested by Nava Diosdado et al. [16] and De Hoyos et al. [12]. The instrument allowed us to identify values and elements of the professional trajectory in the following areas:

- Elements that influenced the interviewee’s vocational choice: For this we took a brief life story. In this area we also gathered demographic data such as age, sex, role in the healthcare system, centre of affiliation, year of entry to the medical centre, location (municipality/borough).
- Career trajectory: We wanted to know how the interviewee got to where they are. We gathered academic information, which included: location, institution, year of graduation and degree obtained as well as whether or not they had received any further certification. We also asked after the year of residency, place of birth, family context, marital status and whether they subscribe to a religion.
  Afterwards, the interviewing method changed to be open-ended while trying to lead the interviewee to indicate what drove him to choose the profession he is in. The specific *contents* which indicate the reasons why they chose to study medicine in order to “serve”, “help others”, “know man” started being identified.
- Work identity: We wanted to know the values with which the interviewee works in order to understand the attitude in their relationships within the workplace. We asked after their schedule, calendar, whether they had missed work (and why), if they identified any particular work-related risk. Additionally, we asked for their opinion regarding the difficulties they face daily and the way in which they are resolved.
- Doctor-patient relationship: we were interested in understanding the manner in which they interact, as well as the values which are generated in a hospital setting. We explored the preference for certain kinds of patients, the number of patients they see in a day and the time they take to tend to them. To study the kind of relationship, we asked whether they could usually remember a patient’s name and the way in which their condition has evolved; we also looked into what kind of relationship they form (authority, parental, friendly etc.). It was also attempted to register whether there was acceptance or rejection towards certain patients and certain perceived social sectors such as: race, ethnicity, sexual preference and social status. Lastly, we identified how the doctor relates information to the patient and how he decides to get involved in the family.
- Future professional projection: By asking the interviewee about the way in which they see their future, we were hoping to get data that would shed light on their values, professional performance and, personal sphere

The final part of the interview included the presentation of vignettes with clinical case studies; this, coupled with the open-ended questions is very important in order to identify perceptions, attitudes and ethical values, this strengthens our qualitative investigation

Even though the data that can be extracted from interviews are vast, we are interested in showing the technical-methodological procedure used to identify the qualitative elements; for instance, the data obtained about values and career trajectory are related to a social and historic context. In short, the ethnographic device allows adding context to the social agents in order to establish rank, levels, tendencies, significance, variations, etc.

### Participants and data collection

Mexican healthcare personnel with current active practices in several clinical areas of a public hospital, including physicians, nurses, and social workers, were invited to participate, at no cost, in an online course on clinical ethics from September 2009 to February 2010. This Continuing Medical Education (CME) course in Clinical Ethics was executed as described by Altamirano et al. and included topics pertaining to the patient and human dignity, healthcare professional-patient relationship, clinical ethics committees, and methodologies for ethical discernment [11]. The qualitative study was performed following the guidelines established by the Cross-functional Group in Clinical Ethics [11] and was supplemented by a hermeneutic analysis, together with the creation of new codes (see below).

30 semi-structured interviews were presented and contrasted adopting an ethnographic approach—taken to mean a descriptive technic used in social theory—, the participants are healthcare professionals who are active in various public hospitals in Mexico. The universe of our study are doctors, nurses, clinical support staff for diagnosis, social workers, and eventually administrative personnel who took part in a distance learning course on clinical ethics in 2009. 15 healthcare professionals were randomly chosen for this study.

Individual face-to face, semi-structured interviews were performed, each following an interview guide. Each interview was directed by anthropologists trained in qualitative investigations, the interviews were recorded and the contents was inputted into an analytic instrument that allowed us to identify the values and career path of the interviewee. The information was codified using as a starting point the content analysis technic created by De Hoyos et al. [12]

The interview maintained the anonymity of the interviewee and took place between September 2009 and February 2010 before the educational intervention and after the educational intervention [16]. The interviews lasted approximately 1.33 hours. These were carried out face to face, the better to build rapport and horizontal conversation in the room. The interviews were recorded and then transcribed by the anthropologists who carried out the interview.

The study was duly sanctioned by the Research Ethics Committee of the Hospital. All participants received written and verbal information about the study and signed a letter of informed consent granting the authors permission to use and publish the data and results obtained; thus maintaining informed consent.

### Analysis of the transcribed interviews

The transcribed interviews were codified and analysed by at least two members of the trans-functional group (which is made up anthropologists, philosophers, literature majors and doctors). The data went into Atlas. Ti. 6.0 where it was codified and the emerging themes were identified. The results were discussed and interpreted by the entire trans-functional group during focused work meetings.

The analysis was carried out following the method described by De Hoyos et al. [12]. All data was analysed in five stages: first, familiarization with the data through listening and immersion in the raw data several times; second, identification of a framework; third, coding; fourth, charting; fifth, interpretation. An axiological framework was developed in line with Schwartz’s [17] work values as well as Pellegrino [2], Oakley & Cocking’s [18] findings regarding the virtues and vices of healthcare professionals.

The most notable emerging themes were representations of medical practice. Beliefs, desires, meanings and the structure of their axiological interactions in clinical practice were studied. 100 codes were grouped according to the following subjects: life history, workday, ethical discernment, doctor-patient relationship, medical procedures, decision making, ethics committee, and future expectations of healthcare professionals.

### Hermeneutic text analysis (Fig 1)

A hermeneutic approach to textual analysis was employed as a mean to attain a deeper understanding of the interview transcriptions [19]. Specifically, we drew from the theory of interpretation propounded by the French philosopher Paul Ricœur. This type of hermeneutics comprises a dialectic process that progresses from the whole to its parts and from comprehension to explanation, thus producing a circle of interpretation that allows understanding to be improved and deepened on a continuous basis [20,21]. Moreover, Ricoeur’s compromise between the ontological and methodological dimensions of the interpretative process renders his approach an appositive tool for a pluralistic analysis such as ours [22,23].

**Fig 1:**
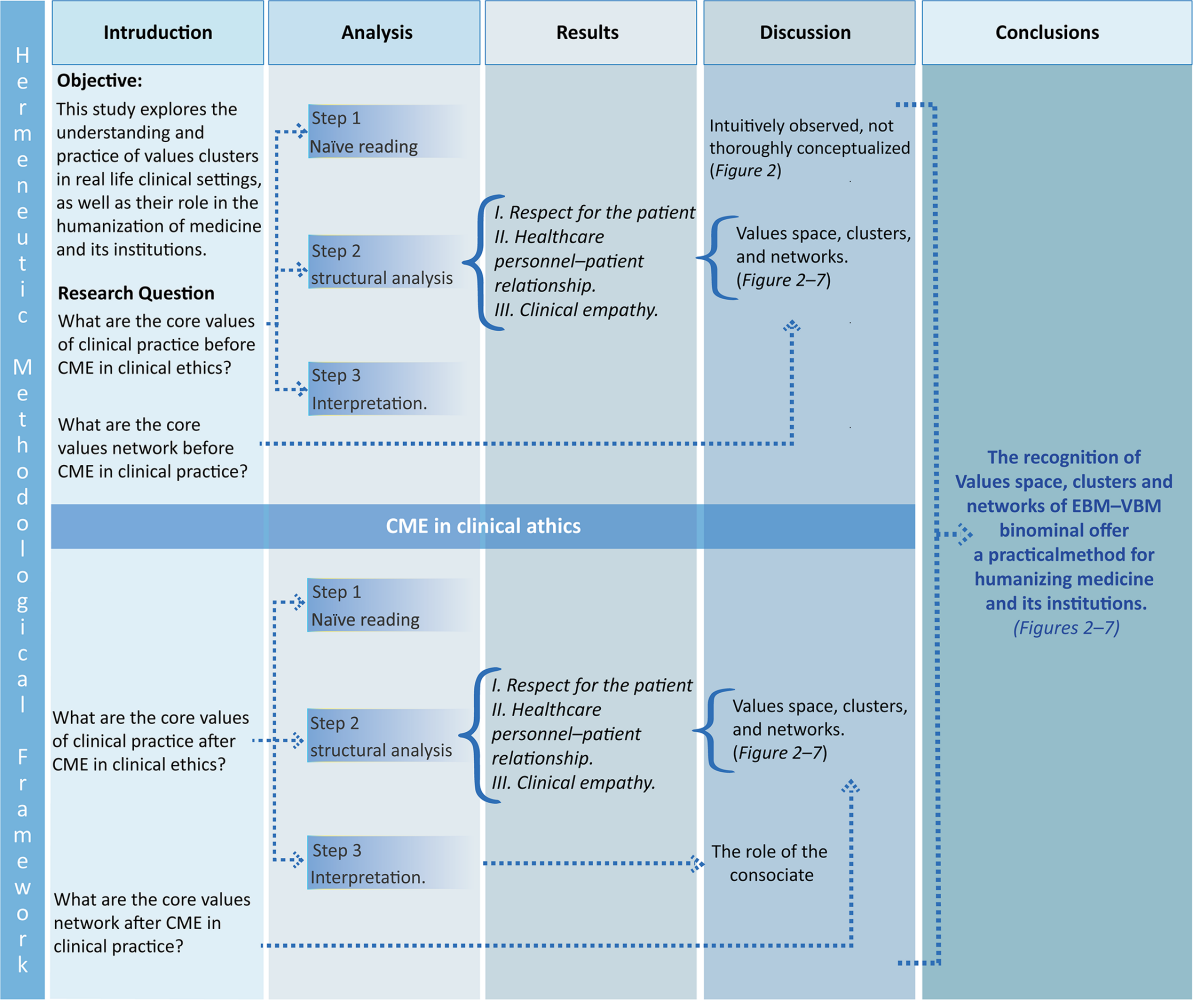
Hermeneutic methodological framework. In step 1, we used “naive reading” to build up semantical network to examine the axiology of clinical practice. In step 2, we used structural analysis of semi-structured interviews (SSIs) to examine. The core values of clinical practice. In step 3 we used interpretation analysis of SSIs to explore the axiology (Values space, clusters and Networks) in clinical practice prior to conducting the CME in clinical ethics. After research questions were examined, these results were integrated based on the mutual validation model, which regards the search for convergent findings as validity indicators.We explored potentially strong connections between EBM and VBM using qualitative results, while we inferred the extent of the benefits of novel Networks and clusters. After conducting the CME in clinical ethics intervention, we repeated the analyses (steps 2 and 3), and the full results were integrated.

Our analysis thus encompasses three stages. The first corresponds to what Ricoeur describes as a ‘naïve reading’, an initial appraisal of the interview transcriptions that is as open-minded as possible, which allows a holistic understanding. As a basis for future analysis, qualitative descriptions are established from the cases presented in the interviews and from significant shared patterns. A structural analysis is then performed, which involves codifying the interview transcriptions and dividing them into their component parts to identify significant content. Each sense unit is condensed, abstracted, and organized into meaning units, sub-themes, and themes, We called a sense unit, the meaning that is condensed from a hermeneutic interpretation. Thus, sense units have an excess of meaning, which allows us to have shared meaningful patterns through a heuristic function. As a result of this process, the naïve reading is either confirmed or rejected. The final stage is aimed at attaining a more comprehensive understanding of the interview transcriptions. At this point, transcriptions are once again considered as a whole, but the interpretation now takes into account the naïve reading and the structural analysis, research questions, and research pre-understandings.

## Results

### Naïve reading: Intuitively observed, not thoroughly conceptualized

**Fig 2:**
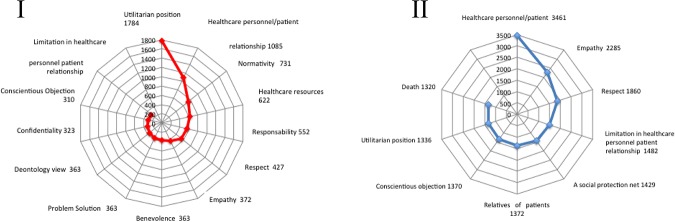
Value semantic networks. Keywords were identified using the Atlas.ti 6.0 software. The words were sorted according to the frequency of their appearance in the interviews. The cut-off point, which divides the set of words into high-frequency and low-frequency groups, was identified. Radial graphs explaining the frequency of appearance were created with MS Excel 2007. The upper left-hand side (I) shows the most relevant values that are consistently mentioned and discussed by the participants prior to the CME intervention on clinical ethics. The lower right-hand side (II) shows the most relevant values that are consistently mentioned and discussed by the participants following the CME intervention on clinical ethics.

The three-step interpretative process of the interview transcriptions recorded before and after the CME course reveals the system of values that is at work among active clinicians and notes new horizons for its expansion. The naïve reading of the interviews suggested that prior to the CME course, a number of values appeared to be somewhat puzzling to healthcare personnel. Many of them were intuitively observed but not thoroughly conceptualized (Fig 2). In terms of the types of relationships established between healthcare professionals and their patients, we noticed that before the CME course, the professionals who are empathic towards the patients tend to maintain what can be described as “medical distance”, appealing more to their professional training than their inner feelings. When terminal patients arrive at the hospital, healthcare personnel are aware that the person is in pain and that the disease causes suffering and depression, but they describe these cases in a mechanical and dispassionate manner. They look after their patients and display a benevolent attitude toward them, but this seems to stem merely from a sense of duty.

Pitfalls in the relationship between healthcare professionals and patients are frequently mentioned in cases where patients do not follow doctors’ orders. In most occasions, it was noted that after an initial failure to follow their treatment plan, the patients returned to their physicians with a more compliant attitude, hoping that the healthcare professionals would understand. Contrary to these patients’ expectations, however, healthcare personnel—as reflected by pre-CME interviews—often reacted in an outwardly paternalistic fashion while feeling inwardly annoyed at and frustrated by this behavior (Fig 2).

### Structural analysis: Values space, clusters, and networks

**Fig 3:**
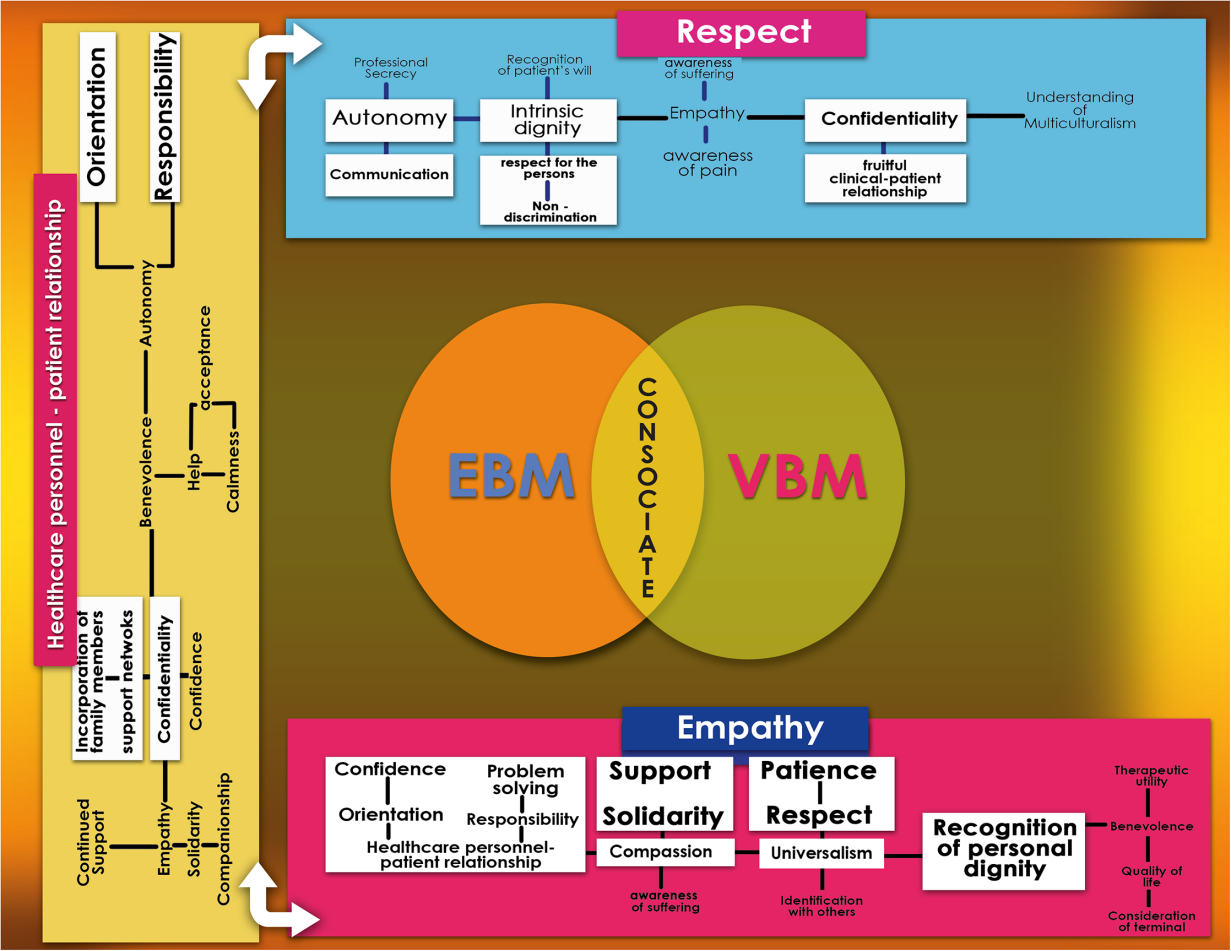
“Values space, clusters, and networks”. Empathy Empathy is a main value imbued in a larger axiological cluster, in which the central axis consists of **compassion,** universalism, **recognition of the person and their dignity**, benevolence, and a **positive clinician-patient relationship.** Originating from this main axiological pillar, we found **support, solidarity,** affective relationships, **patience, respect,** identification with others, therapeutic utility, improvement of the quality of life, **orientation, efficient problem solving, confidence,** and **responsibility.** Respect The axiological spinal cord of respect comprises **intrinsic dignity,** empathy, **autonomy**, **confidentiality** and understanding of multiculturalism. From these values, recognition of the patient’s will, **respect for the person**, **non-discrimination**, awareness of pain and suffering, professional secrecy, **fruitful clinician-patient relationship** and **communication are derived**. Of the above qualities, those marked in bold font are the values to which clinicians most frequently alluded before the CME intervention, although they did not necessarily name them as such. Those listed in regular font were either inexistent or barely perceptible before the course but began to appear in the second set of interviews.

During the structural analysis, we identified that values in clinical practice (both ethical and epistemic) are recognized, promoted, strengthened, and developed freely in what we call a clinical values space, which encompasses the manifold aspects of the biomedical, ethical, social and economic dimensions of everyday health care. In this stage of the analysis, three main values were identified as the most frequently employed by interviewees before and after CME training—namely, respect for the patient, healthcare personnel-patient relationship, and clinical empathy (Figs 2 and 3). Values that are interrelated and/or collateral to these main values emerged, as detailed in the following sections. All three are interdependent with one another and establish dynamic relationships both among themselves and with the related values. Values group and regroup in clusters, which allows for adjustment through a process of natural selection that occurs in the unique and singular moment of interaction between clinicians and patients. It is precisely at this point in time and space—for instance, when diagnosing, using a medical device, or drafting a case history—that clusters of values undergo adjustments and enhancements based on relevance. Our main objective was to unpack the dynamics of the interaction between the clusters and networks of the recognized values and then trace their influence upon the consolidation of the EBM-VBM binomial.

#### 1. Respect for the patient before CME

At the onset of our study, we considered a standard definition of respect as ‘consideration for others’. After a structural analysis of this set of interviews, however, we uncovered other sense units that were associated with this value. In medical practice, the idea of respect is used in a vague manner because it appears linked to a large number of various values. For example, “respect” occasionally appears in relation to human life and dignity, but at other times, it appears related to respect for autonomy. Respect also denotes respect for cultural differences, privacy, and confidentiality [24] Among these, respect for doctor-patient communication, autonomy, and confidentiality emerged. For instance, although physicians attempt to assume the role of guide and advisor, in cases when patients decide not to communicate their illness to their families, they tend to respect the patients’ autonomy and desire for confidentiality [25].

As one interviewee stated,

‘information is to be kept between the doctor and the patient, period. If the family member asks me for information about the patient but the patient is conscious and has asked me not to disclose any relevant information, I don’t’.

Most healthcare professionals in pre-CME interviews declare that confidentiality is to be respected as long as the patient is competent and patient autonomy is seen as constructive.

We also observed that healthcare personnel associate the idea of respect with non-discrimination [26]. The interviewees stated that regardless of their social class, sexual preferences, legal history, and national origins, patients must be treated with respect because dignity is intrinsic to every person. When asked about the approach to follow when treating a patient who is a dangerous criminal, for example, a doctor stated that

‘irrespective of whether the patient may have killed or injured someone, he is a human being [… and] as a person must be cared for.’

Another crucial aspect linked to respect stemming from the non-discrimination aspect is that healthcare professionals consider the patient to be a person, regardless of the condition that caused him/her to seek medical care.

**Fig 4:**
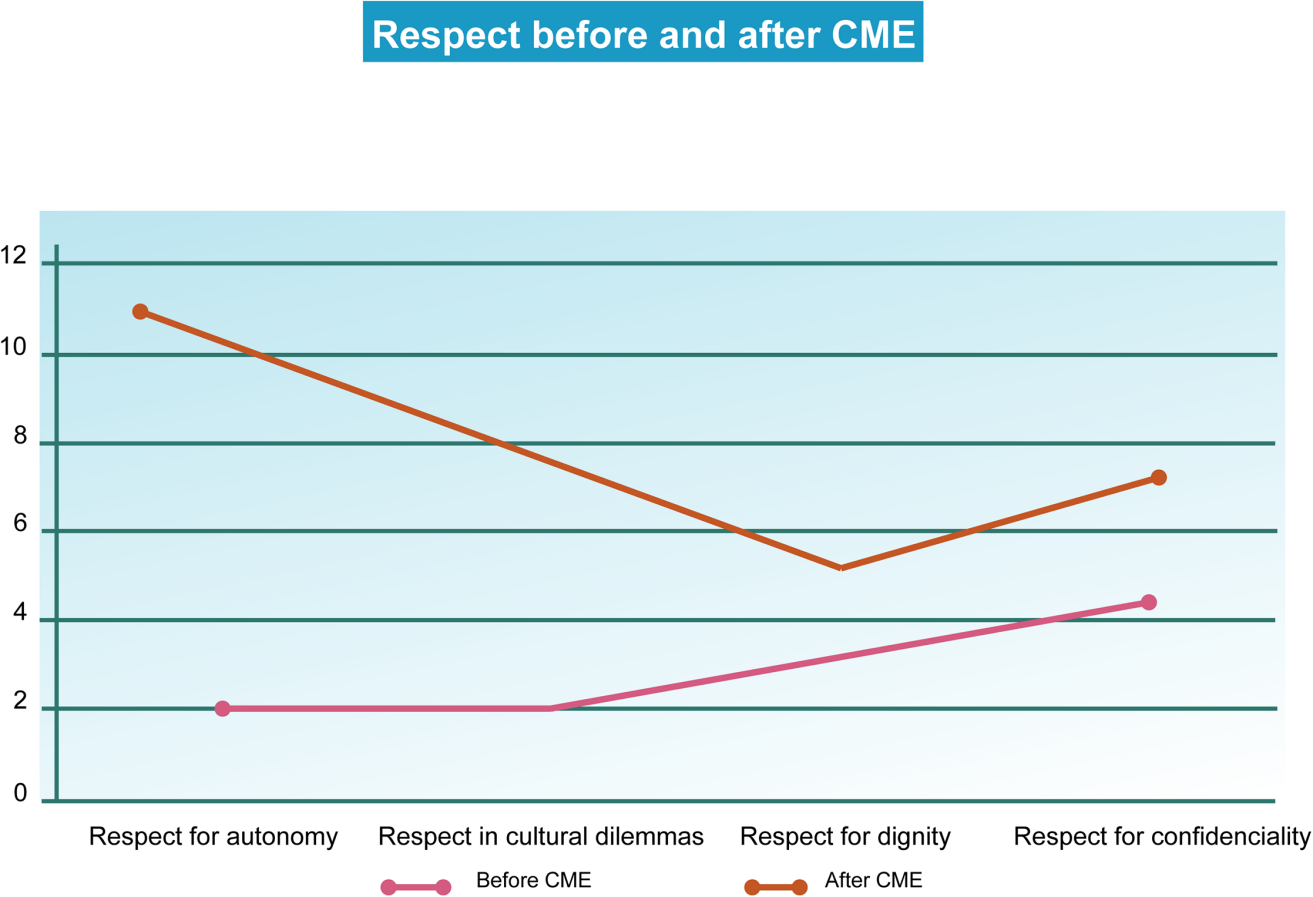
Respect. The keywords were identified using the Atlas.ti 6.0 software. The words were sorted according to the frequency of their appearance in the interviews. The cut-off point, which divides the set of words into high-frequency and low-frequency groups, was identified. The graphs explaining the frequency of appearance were created with MS Excel 2007.

Other sense units that are intuitively linked to respect by interviewees are already apparent at the pre-CME stage. These include the recognition of a patient’s intrinsic dignity and empathic responses to the patient (Figs 3 and 4).

*1*.*1 Respect for the patient after the CME course*

We found that there are a greater number of references to patient autonomy post-CME, which indicated that the healthcare personnel were able to construct a more robust awareness of the patients’ capacity for self-determination when presented with different options. Similar to what was observed from pre-CME transcriptions, in the second round of interviews, respect for autonomy was found to be linked to the requirement for professional secrecy. As long as a patient is both socially and legally competent and the decisions that he/she makes do not affect third parties, confidentiality is to be upheld (Figs 3 and 4).

In contrast to the first round of interviews, however, healthcare personnel who were interviewed after the CME course showed a significantly higher degree of empathy for the suffering of others [27]. Additionally, autonomy was now distinctly interrelated with issues arising in IPSD. For example, if a suffering patient opted to refuse treatment, healthcare professionals post-CME appeared to be much more inclined to respect his/her will. This was also found to be related to a frequent ethical dilemma regarding the use of euthanasia and orders to not resuscitate.

As one interviewee asserted,

‘euthanasia should be accepted because there are many times when the patient is suffering a lot […] I think you have to respect what he says, respect his autonomy.’

Similarly, respect was linked not only to confidentiality and the pain felt by others but also to cultural dilemmas (Fig 2). Compared with the first set of interviews, we noted post-CME that a greater degree of patient-healthcare professional solidarity was appended to respect. After taking the CME course, healthcare personnel sought to negotiate the differences between distinct cultural traditions and discuss them to strengthen the relationship with their patients and improve communication. One interviewee commented,

I used to think that if the patient refuses to be transfused and this procedure was necessary, if I am a doctor, I must do the procedure because I studied medicine to save lives, but this changed with the course. In the course, we talked a lot about respect for patients and respect for their beliefs. This happens very often with people of certain religions, for example, with Jehovah's Witnesses.

As this remark suggests, after participating in the CME course, the interviewee is more receptive to what is meaningful for the individual patient (Figs 3 and 4).

#### 2. Healthcare personnel-patient relationship before CME training

Doctors and other healthcare personnel pre-CME were frequently observed to assume the role of advisers, particularly when the patients reject treatment. A common example described was patients who refuse blood transfusions on account of their religious beliefs. In most cases, it was reported that the patients were to be advised and informed about the importance and necessity of accepting suitable treatment. However, if the patient persists in his/her refusal, having complied with his/her legal obligation to counsel him/her, the physician is also legally obliged to accept the patient’s decision. The doctor is then to take steps to protect him/herself from possible future legal action.

Another participant stressed the importance of developing a proper relationship with the patient on arrival:

‘I try to introduce myself so that he’ll get to know me, to start a relationship indirectly.’

This is related to the understanding that once a rapport has been established, the doctor can counsel the patient better and identify the best solution for his/her specific illness or condition [28,29]. Listening to the patient becomes the main priority [30]. The above-mentioned interviewee narrates a case in which a female patient in need of dialysis due to kidney failure, which she refused to allow. The physician’s first task was attempting to convince her of the need for dialysis:

‘I spoke to her for maybe three days, telling her that it was the best option for her to be able to go on with her life, although maybe not with the same quality of life, but that there were alternatives, that she shouldn’t give up.’

The interviewee does not disclose the patient’s ultimate decision; however, in spite of the insistence mentioned, there is no sense of paternalism or authoritarianism in this approach to the patient. Our analysis clearly shows that counseling tends not to be limited to giving advice but also involves the healthcare personnel taking into account the patient’s opinions as factors that influence the course and outcome of the treatment plan [31].

**Fig 5:**
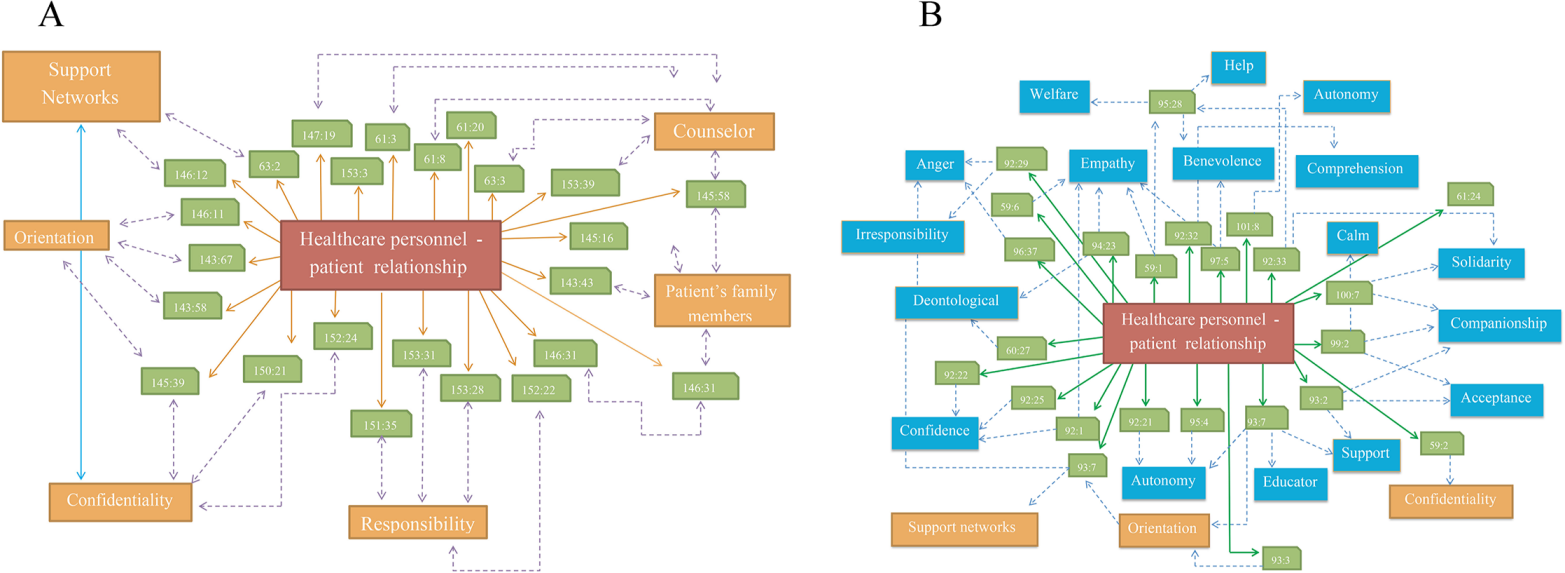
Healthcare personnel-patient relationship value networks before CME training. **A.** Keywords were identified using the Atlas.ti 6.0 software. The words were sorted according to the frequency of their appearance in the interviews. **B. Healthcare personnel-patient relationship value networks after CME training**. Keywords were identified using the Atlas.ti 6.0 software. The words were sorted according to the frequency of their appearance in the interviews.

Another aspect noted in the majority of the examples relating to the healthcare professional-patient interaction was that although the patient alone was responsible for accepting or rejecting the advice given, the network expanded outward to encompass the patient’s family. The resulting axis of communication thus consisted of the healthcare professionals, the patient, and family member(s). Within this context, two values emerge from the structural analysis as being the most often discussed by interviewees: confidentiality and responsibility (Figs 3 and 5A). In cases when a family member wants to know the cause of the patient’s ailment but the latter asks the healthcare team not to disclose it, the value of responsibility is shown to be paramount. First and foremost, the patient’s personal decision and his/her right to privacy are to be respected [32]. Pre-CME participants remarked that this decision can be considered a joint responsibility in the sense that both the patient and healthcare personnel are involved in the treatment and should cooperate in the decision-making process. That the healthcare team is generally aware that the dialog between them and the patient should include a discussion of the pros and cons of various treatment options, benefits, drawbacks, and the degree to which family members should be informed is evidenced in the words of an interviewee:

‘in the event of a contagious disease where people are living in close quarters, well, yeah, we have to explain to the person who is requesting [confidentiality] that he has a significant responsibility toward the other person precisely because of his circumstances’.

Here, it also becomes clear that the nature of the disease is a deciding factor in whether to uphold patient confidentiality, given that this decision may affect third parties.

However, the physician mentioned above also noted that the healthcare practitioner should not assume full responsibility in such cases, as it is the patient who is responsible for following his/her doctor’s advice or not; the patients have the freedom to decide. Similarly, another interviewee explained,

‘I have always been convinced that patients should have a certain amount of responsibility, and if you take all responsibility away from them, they become very coddled.’

As these examples show, responsibility is shared between the healthcare professional and the patient, which is framed by the communication channel (Figs 3 and 5A) [33].

*2*.*1 Healthcare personnel-patient relationship after CME training*

With regard to the healthcare personnel-patient relationship, the structural analysis of the interviews prior to CME showed 1) that communication is based on counseling and guidance; 2) that the family has an important role in the information-sharing process; and 3) that confidentiality and responsibility are prevailing values, although there are limits to confidentiality in certain cases.

**Fig 6:**
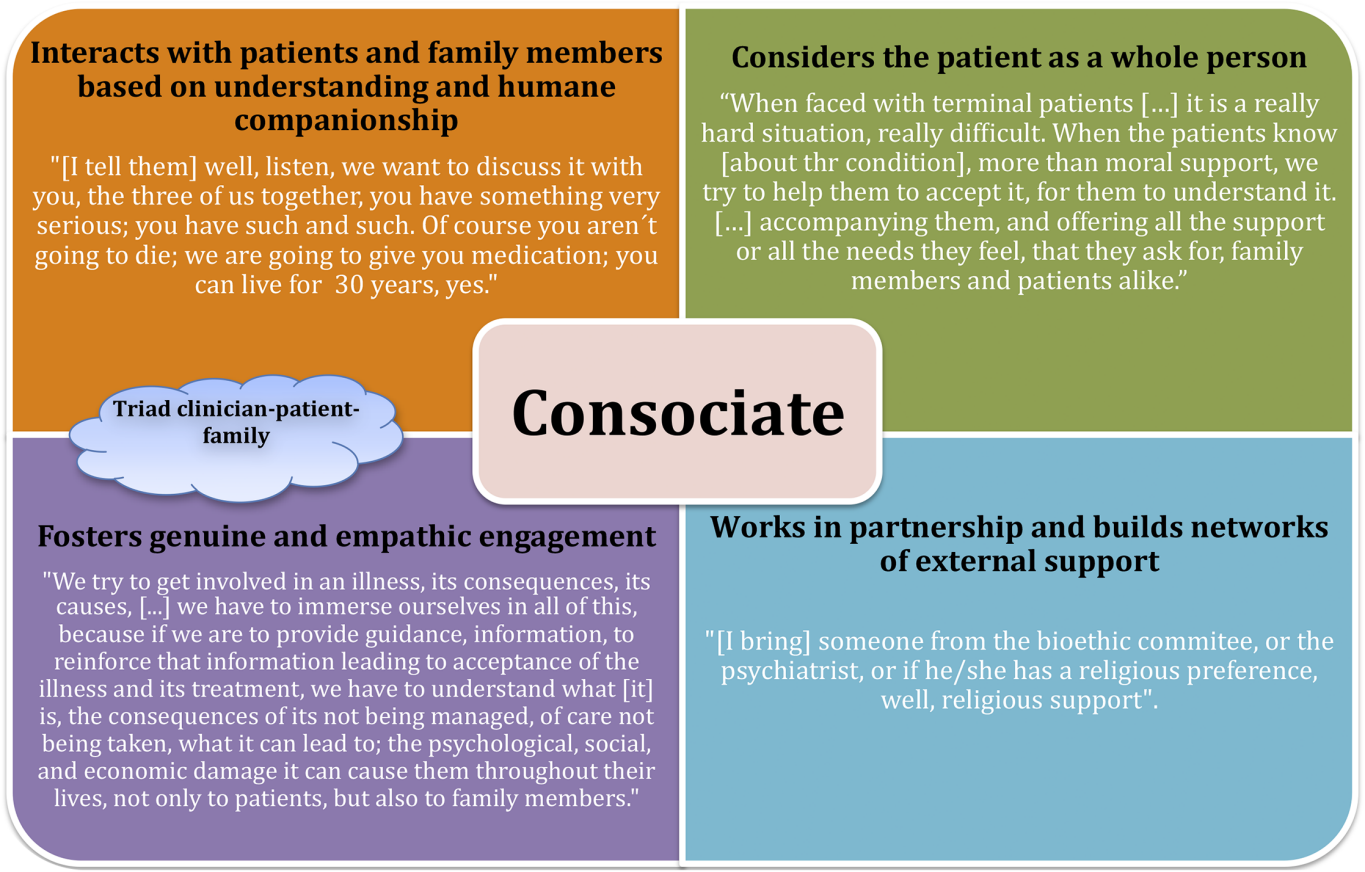
Consociate clinician-patient relationship as cement for the EBM and VBM binomial. Quotations from interviews illustrating its key aspects.

Upon completion of the CME course, however, we observed a remarkable change on the side of the healthcare personnel regarding methods of interaction and communication with patients, family members, and social networks (Figs 3 and 5B). There was a shift from the earlier role of the healthcare professional as a paternalistic educator and advisor that we called the *healthcare professional as a consociate*; consociate refers to the idea that the rapport between patients and healthcare professionals is established in a cooperative relationship. a role in which the caregiver displays a genuine and empathetic cooperative association with the patient and his/her family to identify and achieve agreed-upon goals that fully respect the patient’s needs, wishes, and worldview (Fig 6).

Our appraisal of the opinions stated post-CME reveals that the complexity of the interviewees’ conceptualization of healthcare professional-patient communication developed more deeply. Post-CME, it can be observed that interviewees improved the effectiveness of their interaction with their patients. In addition, there is a shift from focusing primarily on a person’s clinical condition to considering the person as a whole. Although the role of advisor continues to be an important function for the healthcare personnel, the new role of *the healthcare professionals as a consociate* and an educator have been incorporated into the communication paradigm. This modification is evidenced by the comments of one interviewee who was discussing some of the challenges presented by patients with terminal diagnoses:

When faced with terminal patients […] it is a really hard situation, really difficult. When the patients know [about their health conditions], more than moral support, we try to help them to accept it, for them to understand it, […] accompanying them, and offering all the support they need or requirements they have, that they ask for, for family members and patients alike.

Communication, in this case, is based on understanding and human companionship. The analysis reveals that after the CME course, the approach to treatment is explicitly more humane, and the value of empathy is clearly and conspicuously marked. The fact that empathy is much more palpable post-CME training strongly suggests that it is a crucial factor for strengthening the bonds in the healthcare personnel-patient-patient’s family triad (Fig 6).

Moreover, the axis of communication established between the healthcare personnel, patient, and family is fortified via broader support networks within the healthcare team itself. As one interviewee explains,

[W]hen a tough decision has to be made, depending on the case, I have needed outside opinions. […] To give or reinforce information about a patient, which is our job, to explain to the family member about the problems she is facing …. I have to inform the patient, but based on what? […] That’s why I [as a member of the healthcare team] have to be informed about everything, [… and] research together with the people involved in the case in order to make a decision.

Shared interest in learning more about the ailment, then, furthers the bond between patients and healthcare personnel, which enhances communication. This interviewee implies that only after acquiring a wide range of information does he/she feel prepared to make the optimal decision [29]. It is important to note that patients themselves are at the center of this dynamic because the interviewee declares his/her willingness to share the information gathered with the patient and the patient’s family members [30].

Furthermore, it is clear that the healthcare professional makes a much more conscious effort to understand the case thoroughly and to build networks of external support to contribute to the decision-making process. They speak openly about the possibility of bringing external resources (bioethics committee, psychiatric team, chaplain, among others) for added support when a serious development arises. One interviewee powerfully illustrates this implementation of a caring team, stating the types of support that patients can be offered:

[S]omeone from the bioethics committee or the psychiatrist or, if he has a religious preference, well, religious support. Then, sit down, listen, we want to talk with you, the three of us together—you are seriously ill. Of course you aren’t going to die, we are going to give you medication, you can live for 30 years, yes. But sitting down like this, [instead of saying], ‘Oh, how nice for you to have come to my office. Hey, pal, you have AIDS.’ […] I would surround myself with people who could help him at that moment so the shock won’t be so horrendous for him.

The healthcare professional as a *consociate* acknowledges the need to know the patient better to assemble an appropriate support team. By seeing the patient as an integral person, the healthcare team member is now in a position to consider the broader impact of health information, not only on the patient’s physical health but also in other dimensions of the patient’s life [34]. The *healthcare professional as a consociate* can better identify the moral, economic, and social implications of the disease process for the patient’s health and wellbeing (Fig 6). One interviewee describes this as follows:

We try to get involved in an illness, its consequences [and] its causes, because we have to immerse ourselves in all of this, because if we are to give guidance, provide information, reinforce that information, leading to the acceptance of an illness and its treatment, we have to understand what diabetes is, the consequences if it isn’t managed, if care isn’t taken, what it can lead to, what psychological, social and economic damage it can cause them throughout their lives, not only to the patient but also to family members.

Another interviewee speaks of the need for doctors to show sensitivity to the patient’s condition, consider their words carefully in advance and think about the repercussions derived from the way in which news is presented to a patient and his family in terms of acceptance or denial of death, suffering, illness, and pain. A sensitive approach has the added advantage of helping the empathic healthcare practitioner cope with his/her own feelings about the situation [33,35,36]. This happens, for example, when relatives are given information on the status of a terminal patient.

One interviewee declares,

‘especially if the doctor has the tact to tell them, to explain to them, then they begin […] constructing the idea that sooner or later the patient is going to die. So that helps the family member […] and it helps us, doesn’t it?’.

In terms of axiology, the network is now more complex and encompasses a wider range of values such as comprehension, solidarity, companionship, confidence, autonomy, beneficence and empathy, all of which substitute for the prior anti-values. Furthermore, instead of showing indifference or maintaining a rigid professional distance, the health professional exhibits a renewed, healthier, and more humane approach to communication after CME training with empathy as a central value (Figs 3, 5B and 6) [36,37].

#### 3. Clinical empathy before CME training

Within the framework of the health professional-patient relationship, empathy consists in a vicarious affective reaction that stems from the perception and reflection by the healthcare professional of the emotional state or situation of a patient [38]. Our analysis of the first set of interviews shows that the healthcare personnel’s behavior indeed corresponds to the definition of empathy, as they *affectively participate* in the reality of their patients. This, however, is shown mainly with regard to the specific illness in question. As one interviewee comments, ‘in life, we can’t be so heartless’. However, it is important to note that per the strict definition of the term, empathy implies not merely being sensitized to the patients’ pain or suffering but vicariously *feeling* upon reflection on his or her emotions, whereas the compassion (etymologically closer to sympathy) that healthcare professionals develop as a result of their training is often of a different nature and is limited to the accompaniment and sharing of the suffering patients who are under their care [39].

**Fig 7:**
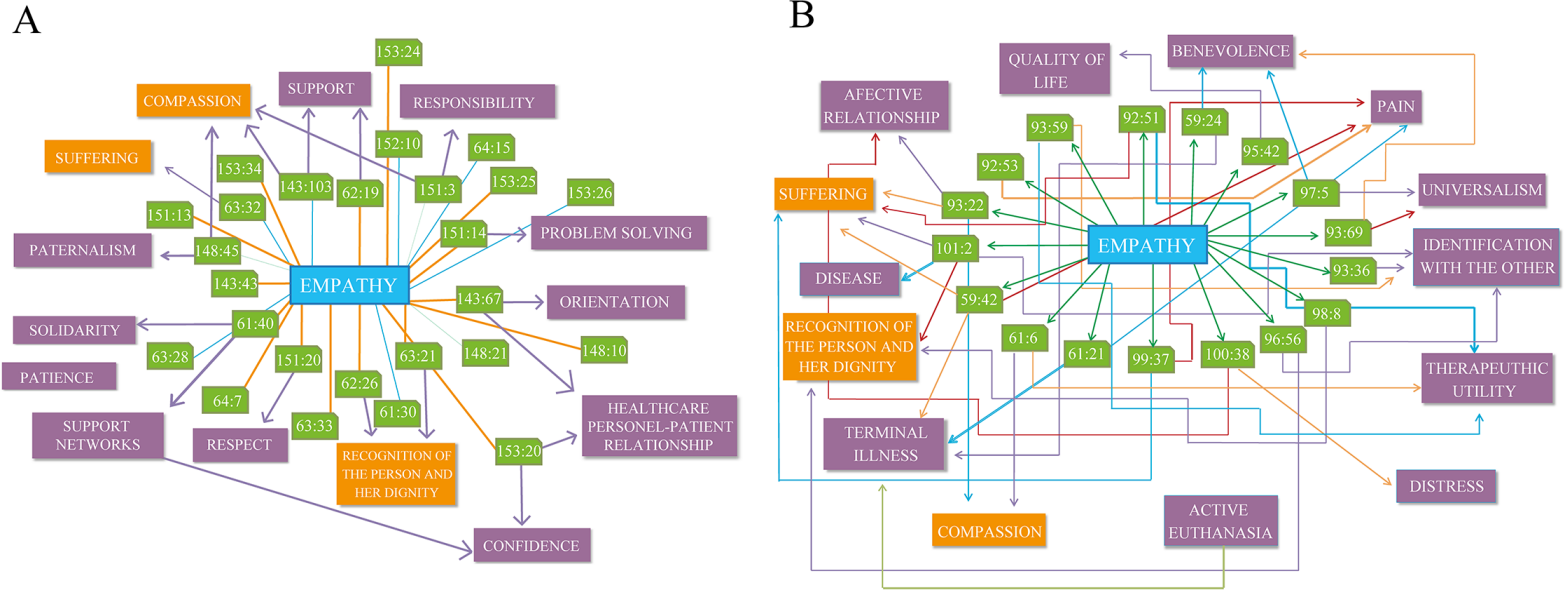
Empathy value networks before CME training. **A.** The keywords were identified using the Atlas.ti 6.0 software. The words were sorted according to the frequency of their appearance in the interviews. **B. Empathy value networks after CME training.** The keywords were identified using the Atlas.ti 6.0 software. The words were sorted according to the frequency of their appearance in the interviews

After an in-depth analysis of empathy and its relationship to other values, the binomial of empathy–compassion stands out as the most ubiquitous and mainly appears in the context where medical attention, duty, and the responsibility of the healthcare practitioner come into play together. Healthcare providers try to provide ‘quality and warmth’ in their practices, and they derive pleasure and satisfaction from seeing that a patient is improving after a difficult period. Thus, empathy is also shown to go hand-in-hand with support and understanding (Figs 3 and 7A).

As one healthcare professional explains, when a transplant is performed successfully,

‘[the patient] will never forget it. I tell her, ‘Take good care of yourself! Take good care of yourself!’ These are things you remember and you say, ‘Oh, isn’t it great? Isn’t it? Doesn’t it make you feel good?”

However, healthcare personnel often mention that they are required to maintain a ‘professional distance’ so they do not become overly involved with the patients.

Finally, empathy is not only shown to patients but also to their family members, who are the patient’s main source of support and his/her primary companions.

One interviewee speaks of the importance of this, declaring that when

‘there’s a lot of empathy for family members […]; you make them see that these procedures, such as the amputation of a foot, has benefits in the end […] it has a reason, a cause’

(Figs 3 and 7A).

*3*.*1 Clinical empathy after CME*

The analysis of the interviews following CME suggests that empathy remains prominent as a central value. Participants’ understanding of empathy, however, has undergone significant changes (Figs 3 and 7B). Post-CME, the compassion expressed is not merely that of a healthcare provider who, in keeping with his training, dutifully offers care and accompaniment during times of suffering. In post-CME interviews, there is evidence of greater sensitivity being shown for the suffering and pain experienced by the patients and the situation that the family faces. Significantly, interviewees no longer allude to the notion that professional distance should be maintained. A qualitatively different approach is evident post-CME, whereby healthcare providers identify themselves with patients and their families *first and foremost* as people rather than solely as part of a healthcare provider-patient capacity. Aspects such as suffering, pain, quality of life, identification with the other, and patient autonomy are evidenced more after CME training than they were before [40].

Also relevant to this analysis is the fact that healthcare team members post-CME are more aware of the will and decisions of terminal patients (Figs 3 and 7B). As a consequence, they make a greater effort to show compassion, recognize their pain, respect their intrinsic dignity and autonomy, and acknowledge that receiving treatment is not necessarily equated with having an acceptable quality of life. As one interviewee describes it,

[W]hen something hurts you, multiply it by a thousand and you’ll see things from the point of view of a cancer patient, right? Imagine the pain you feel; the patient feels it every day; not even morphine makes it go away, nor will the best medicines make it go away either.

Similarly, in cases when mechanical ventilation is necessary to prolong life, if terminal patients want to allow themselves to die to end their intense pain and suffering, healthcare providers tend to respect their autonomy. In the words of one interviewee describing the use of a respirator on a terminal patient,

[N]o, well, it’s artificial. I believe that whenever there’s no sign of life, no brain activity, and that being hooked up doesn’t make sense, it doesn’t make sense to keep him like that. Yes, it’s life, but that life is suffering, you know, so even if he can’t express himself or anything, he is suffering. In that case, I don’t think it’s humane; from my point of view, it isn’t humane.

### Comprehensive understanding: The role of the consociate

For our final, comprehensive examination of the results prior to the CME, the most salient values that emerge are threefold: confidentiality, responsibility, and patient advisory (Fig 5A). After CME training, a wider spectrum of values is represented, including wellbeing, beneficence, trust, autonomy, support, help, solidarity and understanding (Fig 5B).

The analysis of opinions post-CME training revealed a remarkable development of the conceptualization of the key components of the clinician-patient relationship. Participants stressed the importance of fruitful interaction and more efficacious communication between clinicians, patients, family members, and other social networks. Crucially, there was a shift from the earlier role of the clinician as a paternalistic educator and advisor to what we call the clinician as a consociate, a role in which a genuine and empathic cooperative relationship between all individuals involved in the treatment emerges. Communication with patients and family members is then based on understanding and humane companionship. One of the most relevant changes after the CME course on clinical ethics was the conception of their role as part of this consociation, which means consolidating the triad of clinician-patient-family, in which all participants can identify and achieve agreed-upon goals that are clinically pertinent and respectful of the patient’s needs, wishes, and worldview (Figs 3 and 6). This view was confirmed by the intense interactions found in the analysis of these patients, their families and clinicians.

In contrast, although the healthcare personnel had intuitive conceptions of autonomy before CME training, which led to a degree of mutual distrust between healthcare professionals and patients, after CME training, this autonomy was re-conceptualized and better utilized in clinical practice. This is an empirical demonstration that the principles of care can be too strictly applied. The application of autonomy, for instance, may cause the doctor-patient relationship to become exceedingly restricted by legal considerations. What was initially a principle aimed at protecting the patient may end up being distorted into fostering a defensive practice [25]. As Fulford, Peile & Carroll note, ‘regulatory ethics have inherent limitations with regard to individual clinical decisions. In the first place, there is the risk of defensive practice, a risk that has been much increased in recent years by the quasi-legal model of medical decision-making’ [41].

Important changes were also observed concerning the value of empathy. Post-CME, this value was reconsidered and enlarged. This was shown to have a significant impact on the way healthcare personnel understand the multifaceted world of experience of each patient’s day-to-day reality. A better contextualization of empathy helps remind healthcare professionals that each patient is unique and that his/her suffering is not a universal experience that can be fit into preset molds.

Prior to CME, empathy was a recurring value that was expressed mainly as compassion, solidarity and responsibility (Fig 7A) [41]. However, this attitude of ‘putting oneself in the shoes of others’ was something that healthcare professionals prior to CME training identified as a *duty*. After CME training, the healthcare personnel were more receptive and better internalized the virtue of empathy (Fig 7B). This reconceptualization drew closer to a new type of respect for patient dignity and autonomy in the healthcare decision-making process. Empathy and compassion were notably more directed towards ensuring a patient’s wellbeing and avoiding unnecessary pain and suffering. Given that empathy also expresses itself as the desire to better understand a patient’s thoughts and feelings as being about ‘grasping the subjective experiences, wishes, feelings, opinions, thoughts and intentions of another person. […] Placing what the other person is saying in a cultural tradition or a historical horizon will not be adequate or sufficient’ [42], it also implies the virtue of discernment: ‘the person of discernment is disposed to understand and perceive what circumstances demand in the way of human responsiveness’ [43]. As with the case of autonomy, a caveat to bear in mind is that the healthcare professional must not go to the opposite extreme of over-identifying with patients. Empathizing does not imply giving oneself over entirely to the suffering of the other; it means understanding the world from the point of view of the patient. The form of empathy internalized by healthcare professionals after CME training supposes a change from acting out of a sense of duty to building more consociate relationships and bridging the gap between VBM and EBM.

## Discussion

In their daily work, healthcare professionals rely on both tacit and explicit ethical values. These ought to be brought to their attention so that, by being consciously aware of them, they can work to potentiate them, which means that all of the values that healthcare personnel already have can and should be addressed, refreshed, and reinforced by means of continuing education (Figs 1–7) [11].

The Healthcare personnel-patient relationship is at the heart of, the binomial EBM-VBM through its development the epistemic goals of learning about a patient’s health problems, as well as the ethical obligations that arise when a person in need of assistance is before someone who can provide it (Fig 3). Therefore the Healthcare personnel-patient relationship functions as both a means and an end, inasmuch as it does not only describe an institutional state of affairs, but rather a set of values and obligations that are to be met. Since it functions as a system of values, the healthcare personnel-patient relationship should be evaluated as such.

Most (if not all) human relations are profoundly ethical in nature. For example, workmate may in one hand describe a simple relationship with others that attend the same workplace, but it entails ethical obligations that have to be met by those who are in that relationship such as respect and collaboration, because these are features that allow achieving the ends of their mutual work. When we consider a Healthcare personnel-patient relationship, special ends arise since it is a very delicate good that is at stake, i.e. a person’s health. Moreover other aspects of how this relationship is carried out turn out to be also valuable, non-discrimination, attentiveness and respect for cultural differences, meet the obligations of this relationship and are important to achieve the ends of medicine

Our analysis shows that the main values are intricately woven into other sense units and offers a generalized panorama on how CME improves understanding and widens the network of values. From this analysis, indications and possible strategies to promote and expand the practice of VBM can be created.

Working as consociates, clinicians can achieve a fuller and deeper cognizance of the multidimensional aspects of the patients and their families’ experiences around IPSD, thus effectively combining scientific evidence relevant to each case with a humane understanding of the full picture, and we propose that it is precisely in this role that the essential components of the EBM-VBM binomial intersect. The clinician as a consociate is more supportive of the patient’s entitlement to autonomy while also becoming more receptive, thereby developing a closer relationship with the patients and their surrogates. This alteration implies a commendable shift from focusing almost exclusively on a patient’s clinical condition to considering him/her as a person. Consequently, clinicians with a wider system and conceptualization of values can better identify the moral, economic, and social implications of the diagnosis and disease process on the patient’s wellbeing and are therefore more mindful of the impact of the amount of information provided and the way in which it is delivered.

Furthermore, the axis of communication established between the healthcare personnel, patient, and family is fortified via broader networks of support within the healthcare team itself, as illustrated by the relevant quotation for this value in Table 1. Shared interests in learning more about the ailment, then, not only strengthens the bond between various sectors of the clinical settings but also enables more appropriate decision-making.

**Table 1:** Sample quotations from interviews illustrating clinician’s values.

Respect Autonomy	“Information is to be kept between the doctor and the patient, period. If the family member asks me for information about the patient but the patient is conscious and has asked me not to give out any relevant information, I don’t.”
Non-discrimination	“Irrespective of whether the patient may have killed or injured someone, he is a human being [… and] as a person must be cared for.”
Autonomy	“Euthanasia should be accepted because there are many times that the patient is suffering a lot […] I think you have to respect what he says, respect his autonomy.”
Multiculturalism	“I used to think that if the patient refuses to be transfused and this procedure was necessary, if I am a doctor, I must do it because I studied medicine to save lives, but this changed with the course. In the course we talked a lot about respect for patients and respect for their beliefs. This happens very often with people of certain religions for example with Jehovah's Witnesses.”
Fruitful communication	“I spoke to her for maybe three days, telling her that it was the best option for her to be able to go on with her life, although maybe not with the same quality of life, but that there were alternatives, that she shouldn’t give up.”
Confidentiality Responsibility to others	“In the case of a contagious disease, where people are living in close quarters, well, yeah, we have to explain to the person who is requesting [confidentiality] that he has such a great responsibility toward the other person precisely because of his circumstances”
Responsibility of patients	“I have always been convinced that patients should have a certain amount of responsibility, and if you take all responsibility away from them, they become very coddled.”
Support network	“When a tough decision has to be made, depending on the case, I have needed outside opinions. […] To give or reinforce information about a patient, which is our job, to explain to the family member about the problems she is facing […] I have to inform the patient, but based on what? […] That’s why I [as a clinician] have to become informed about everything, [… and] research together with the people involved in the case in order to make a decision about it.”
Empathy Dignity Autonomy	“When something hurts you, multiply it by a thousand and you’ll see things from the point of view of a cancer patient, right? Imagine the pain you feel; the patient feels it every day, not even morphine makes it go away, nor will the best medicines make it go away either.”
Empathy Autonomy	“No, well, it’s artificial. I believe that when there’s no sign of life, no brain activity, and being hooked up doesn’t make sense, it doesn’t make sense to keep him like that. Yes it’s a life, but that life is suffering, you know, so even if he can’t express himself or anything, he is suffering. In that case, I don’t think it’s humane, from my point of view, it isn’t humane.”

The role of the consociate is simultaneously dependent upon and induces the development of a shared framework of values between all three components of the axis of interaction. Thus, a more adequate conceptualization of values engenders the role of consociate, and this, in turn, promotes the expansion of the axiological network in daily practice. The result of these complementary processes leads to a genuine cementation of the two models of modern medicine. For instance, once the constituents and ramifications of empathy are properly appreciated, the clinician-patient relationship flows more naturally, thus propitiating a more humanitarian and compassionate treatment experience that epitomizes the heart and art of medicine while allowing for a more discerning usage of scientific evidence and technological resources. The implications of this are important both for a fuller appreciation of the interactions between EBM and VBM in daily practice and for the design of more effective models of CME that target the values identified as crucial [11,12].

Our study empirically demonstrates the functioning of a values space, the cluster of values and the networks of values on which clinicians rely and put into practice on a daily basis. Unfolding the cognitive representations of healthcare professionals’ own experiences in relation to IPSD, our analysis illustrates ways in which cross-functional educational interventions can lead to demonstrable changes in attitudes and practices. Showing the intricate clusters and networks into which values are interwoven (Figs 3–7), this study suggests that certain aspects in particular should be addressed, refreshed, and reinforced by means of targeted continuing education, namely, appreciation of the personhood of patients, empathetic awareness of their pain, suffering, disease process and the meaning of their existence to themselves and to others and, when relevant, their imminent demise. Keeping these elements in mind alleviates the tendency of mistaking patients for their illness and treating them as objects in need of repair. Finally, but equally important, our paper proposes to add the consociate aspect to the role of the clinician as a desirable goal that would allow a more successful integration of the complementary components of the EBM-VBM binomial (Figs 3 and 6). All of these points offer a practical method for humanizing medicine and its institutions to the field of bioethics.

## Supporting information

S1 Table Coreq check list. Consolidated criteria for reporting qualitative studies (COREQ): 32-item checklist.
(DOC)

## References

[1] Little JM. Humanistic medicine or values-based medicine. what’s in a name? Med J Aust. 2002;177: 319–21. Available: http://www.ncbi.nlm.nih.gov/pubmed/12225281 12225281 10.5694/j.1326-5377.2002.tb04792.x

[2] Pellegrino ED. Professionalism, profession and the virtues of the good physician. Mt Sinai J Med. 2002;69: 378–384. Available: http://www.ncbi.nlm.nih.gov/pubmed/12429956 12429956

[3] Fulford KWM. The value of evidence and evidence of values: bringing together values-based and evidence-based practice in policy and service development in mental health. J Eval Clin Pract. 2011;17: 976–987. doi: 10.1111/j.1365-2753.2011.01732.x 21951930 10.1111/j.1365-2753.2011.01732.x

[4] Fulford KWM. Bringing together values-based and evidence-based medicine: UK Department of Health Initiatives in the “Personalization” of Care. J Eval Clin Pract. 2011;17: 341–343. doi: 10.1111/j.1365-2753.2010.01578.x 21114716 10.1111/j.1365-2753.2010.01578.x

[5] Peile E. Evidence-based medicine and values-based medicine: partners in clinical education as well as in clinical practice. BMC Med. 2013;11: 40 doi: 10.1186/1741-7015-11-40 23414247 10.1186/1741-7015-11-40 PMC3626674

[6] Harman G. Explaining value and other essays in moral philosophy Oxford: Clarendon Press; 2000.

[7] Putnam H. The Fact/Value Dichotomy and the Future of Philosophy In: Marchetti G, Marchetti S, editors. Facts and Values: The Ethics and Metaphysics of Normativity. New York: Routledge; 2017 pp. 27–41.

[8] Brown NC, McGee SJ. Conceptualizing boundaries for the professionalization of healthcare ethics practice: a call for empirical research. HEC Forum. 2014;26: 325–341. doi: 10.1007/s10730-014-9240-x 24973870 10.1007/s10730-014-9240-x

[9] Godbold R, Lees A. Ethics education for health professionals: a values based approach. Nurse Educ Pract. 2013;13: 553–560. doi: 10.1016/j.nepr.2013.02.012 23517926 10.1016/j.nepr.2013.02.012

[10] Manzi A, Magge H, Hedt-Gauthier BL, Michaelis AP, Cyamatare FR, Nyirazinyoye L, et al Clinical mentorship to improve pediatric quality of care at the health centers in rural Rwanda: a qualitative study of perceptions and acceptability of health care workers. BMC Health Serv Res. 2014;14: 275 doi: 10.1186/1472-6963-14-275 24950878 10.1186/1472-6963-14-275 PMC4077561

[11] Altamirano-Bustamante MM, Altamirano-Bustamante NF, Lifshitz A, Mora-Magaña I, de Hoyos A, Avila-Osorio MT, et al Promoting networks between evidence-based medicine and values-based medicine in continuing medical education. BMC Med. 2013;11: 39 doi: 10.1186/1741-7015-11-39 23414220 10.1186/1741-7015-11-39 PMC3606451

[12] de Hoyos A, Nava-Diosdado R, Mendez J, Ricco S, Serrano A, Flores Cisneros C, et al Cardiovascular medicine at face value: a qualitative pilot study on clinical axiology. Philos Ethics Humanit Med. 2013;8: 3 doi: 10.1186/1747-5341-8-3 23531271 10.1186/1747-5341-8-3 PMC3620706

[13] Parker M. Ethnography/ethics. Soc Sci Med. 2007;65: 2248–2259. doi: 10.1016/j.socscimed.2007.08.003 17854966 10.1016/j.socscimed.2007.08.003

[14] Lock M. The Tempering of Medical Anthropology: Troubling Natural Categories. Med Anthropol Q. Blackwell Publishing Ltd; 2001;15: 478–492. doi: 10.1525/maq.2001.15.4.478 11794872 10.1525/maq.2001.15.4.478

[15] Long SO. Life is more than a survey: understanding attitudes toward euthanasia in Japan. Theor Med Bioeth. 2002;23: 305–19. 12516835 10.1023/a:1021243805657

[16] Nava Diosdado R, Flores Cisneros C, Méndez Jiménez J, Serrano Zamago A, de Hoyos Bermea A, Ricco Monge S, et al Valores en Medicina: etnografía de sus representaciones en un hospital de cardiología en México. Cuicuilco. Escuela Nacional de Antropología e Historia; 2011;18: 115–132.

[17] Schwartz SH. Universals in the Content and Structure of Values: Theoretical Advances and Empirical Tests in 20 Countries. Adv Exp Soc Psychol. 1992;25: 1–65. doi: 10.1016/S0065-2601(08)60281-6

[18] Oakley J, Cocking D. Virtue ethics and professional roles. Cambridge: Cambridge University Press; 2006.

[19] Lindseth A, Norberg A. A phenomenological hermeneutical method for researching lived experience. Scand J Caring Sci. 2004;18: 145–153. doi: 10.1111/j.1471-6712.2004.00258.x 15147477 10.1111/j.1471-6712.2004.00258.x

[20] Ricœur P. The rule of metaphor: multi-disciplinary studies of the creation of meaning in language Toronto: University of Toronto; 1978.

[21] Ricœur P. From Text to Action: Essays in Hermeneutics, II. Evanston: Northwestern University Press; 1991.

[22] Geanellos R. Exploring Ricoeur’s hermeneutic theory of interpretation as a method of analysing research texts. Nurs Inq. 2000;7: 112–119. Available: http://www.ncbi.nlm.nih.gov/pubmed/11075108 11075108 10.1046/j.1440-1800.2000.00062.x

[23] Dreyer PS, Pedersen BD. Distanciation in Ricoeur’s theory of interpretation: narrations in a study of life experiences of living with chronic illness and home mechanical ventilation. Nurs Inq. 2009;16: 64–73. doi: 10.1111/j.1440-1800.2009.00433.x 19228305 10.1111/j.1440-1800.2009.00433.x

[24] Beach MC, Duggan PS, Cassel CK, Geller G. What does “respect” mean? Exploring the moral obligation of health professionals to respect patients. J Gen Intern Med. 2007;22: 692–695. doi: 10.1007/s11606-006-0054-7 17443381 10.1007/s11606-006-0054-7 PMC1852905

[25] Barilan YM, Weintraub M. Persuasion as respect for persons: an alternative view of autonomy and of the limits of discourse. J Med Philos. 2001;26: 13–33. doi: 10.1076/jmep.26.1.13.3033 11262639 10.1076/jmep.26.1.13.3033

[26] Fjellstrom R. Respect for persons, respect for integrity: remarks for the conceptualization of integrity in social ethics. Med Health Care Philos. 2005;8: 231–242. doi: 10.1007/s11019-004-7694-3 16215802 10.1007/s11019-004-7694-3

[27] Missel M, Birkelund R. Living with incurable oesophageal cancer. A phenomenological hermeneutical interpretation of patient stories. Eur J Oncol Nurs. 2011;15: 296–301. doi: 10.1016/j.ejon.2010.10.006 21111678 10.1016/j.ejon.2010.10.006

[28] Savory EA, Marco CA. End-of-life issues in the acute and critically ill patient. Scand J Trauma Resusc Emerg Med. 2009;17: 21 doi: 10.1186/1757-7241-17-21 19386133 10.1186/1757-7241-17-21 PMC2678074

[29] Okon TR. &quot;Nobody understands&quot;: on a cardinal phenomenon of palliative care. J Med Philos. 2006;31: 13–46. doi: 10.1080/03605310500499161 16464768 10.1080/03605310500499161

[30] Dörr A. [On doctor-patient communication from an anthropological and social point of view]. Rev meédica Chile. 2004;132: 1431–1436. Available: http://www.ncbi.nlm.nih.gov/pubmed/15693208 10.4067/s0034-98872004001100014 15693208

[31] Evans N, Pasman HRW, Payne SA, Seymour J, Pleschberger S, Deschepper R, et al Older patients’ attitudes towards and experiences of patient-physician end-of-life communication: a secondary analysis of interviews from British, Dutch and Belgian patients. BMC Palliat Care. 2012;11: 24 doi: 10.1186/1472-684X-11-24 23186392 10.1186/1472-684X-11-24 PMC3583811

[32] Frankena WK. The Ethics of Respect for Persons. Minar E, editor. Philos Top. 1986;14: 149–167. doi: 10.5840/philtopics19861428

[33] Polleiro MC. Comunicación médico-paciente. Med Gen. 2005;75: 513–515.

[34] Braddock CH, Snyder L, Neubauer RL, Fischer GS, American College of Physicians Ethics, Professionalism and Human Rights Committee and The Society of General Internal Medicine Ethics Committee. The patient-centered medical home: an ethical analysis of principles and practice. J Gen Intern Med. 2013;28: 141–146. doi: 10.1007/s11606-012-2170-x 22829295 10.1007/s11606-012-2170-x PMC3539020

[35] García M C, Ortega T D. Comunicación en los tiempos de la colera: clínicos y radiólogos. Rev Chil Radiol. Sociedad Chilena de Radiología; 2004;10: 186–190. doi: 10.4067/S0717-93082004000400008

[36] Pedersen R. Empathy: a wolf in sheep’s clothing? Med Health Care Philos. 2008;11: 325–335. doi: 10.1007/s11019-007-9104-0 17926143 10.1007/s11019-007-9104-0

[37] Gair S. Feeling their stories: contemplating empathy, insider/outsider positionings, and enriching qualitative research. Qual Health Res. 2012;22: 134–143. doi: 10.1177/1049732311420580 21873286 10.1177/1049732311420580

[38] Weiner SJ, Auster S. From empathy to caring: defining the ideal approach to a healing relationship. Yale J Biol Med. 2007;80: 123–130. Available: http://www.ncbi.nlm.nih.gov/pubmed/18299724 18299724 PMC2248287

[39] Scott JG, Scott RG, Miller WL, Stange KC, Crabtree BF. Healing relationships and the existential philosophy of Martin Buber. Philos Ethics Humanit Med. 2009;4: 11 doi: 10.1186/1747-5341-4-11 19678950 10.1186/1747-5341-4-11 PMC2733137

[40] van Hooft S. Pain and communication. Med Health Care Philos. 2003;6: 255–262. Available: http://www.ncbi.nlm.nih.gov/pubmed/14620462 14620462 10.1023/a:1025956726573

[41] Fulford K, Peile E, Carroll H. Essential Values-Based Practice Clinical Stories Linking Science with People. Cambridge: Cambringe University Press; 2012.

[42] Alma HA, Smaling A. The meaning of empathy and imagination in health care and health studies. Int J Qual Stud Health Well-being. 2006;1: 195–211. doi: 10.3402/qhw.v1i4.4934

[43] Beauchamp TL, Childress JF. Principles of Biomedical Ethics. New York: Oxford University Press; 2009.

