# Supplementary material for: Today´s medical self and the other: Challenges and evolving solutions for enhanced humanization and quality of care
Source: PLoS One. 2017 Jul 31;12(7):e0181514. doi: 10.1371/journal.pone.0181514 (PMC5536364; doi:10.1371/journal.pone.0181514)
Supplement: S1 Table — Consolidated criteria for reporting qualitative studies (COREQ): 32-item checklist. (DOC) [file pone.0181514.s001.doc]

**Consolidated criteria for reporting qualitative studies (COREQ): 32-item checklist.**

**Today´s medical self and the other: challenges and evolving solutions for enhanced humanization and quality of care**

Perla Sueiras, Victoria Romano-Betech, Alejandro Vergil-Salgado, Adalberto de Hoyos, Silvia Quintana-Vargas, William Ruddick, Anaclara Castro-Santana, Sergio Islas-Andrade, Nelly F. Altamirano-Bustamante, Myriam M. Altamirano-Bustamante

**Domain 1: Research team and reflexivity**

*Personal Characteristics*

1. Interviewer/facilitator Which author/s conducted the interview or focus group? The interviewer were performed by anthropologists coordinated by Myriam M. Altamirano-Bustamante and Adalberto de Hoyos

2. Credentials. What were the researcher’s credentials? Myriam M. Altamirano-Bustamante is a MD and PhD. Adalberto de Hoyos is a PhD in Philosophy.

3. Occupation. What was their occupation at the time of the study? The anthropologists and literature experts were MA students, and one of the coordinator was a postdoctoral researcher, the other coordinator was a full time researcher at the IMSS.

4. Gender. Was the researcher male or female? The anthropologists were 3 male and 3 female. The coordinators one was female and one male.

5. Experience and training. What experience or training did the researcher have? 5 anthropologist have Bachelor in Physical Anthropology and one in Social Anthropology and were coursing Master’s studies in Bioethics, Philosophy of Science and Physical Anthropology. All with 4 years of field research experience. The coordinator have experience in Bioethics, Philosophy and Philosophy of Science research.

*Relationship with participants*

6. Relationship established was a relationship established prior to study commencement? The coordinator (MMAB) had times contact in three different occasions with the participants for the invitation steps, the informed consent step and the day of the interview before CME. The anthropologist meet the participants in the first interview before CME.

7. Participant knowledge of the interviewer

What did the participants know about the researcher? The participants had knowledge of the reasons for doing the research and the scopes of the research.

8. Interviewer characteristics. What characteristics were reported about the interviewer/facilitator? All the interviewer have reasons and interests in the research topic.

**Domain 2: study design**

Theoretical framework

9. Methodological orientation and Theory

What methodological orientation was stated to underpin the study? The theoretical Framework was an amalgam of ethnography -content and interpretation analysis (hermeneutics)-Bioethics.

Participant selection

10. Sampling. How were participants selected?

The universe of our study are doctors, nurses, clinical support staff for diagnosis, social workers, and eventually administrative personnel who took part in a distance learning course on clinical ethics in 2009. 15 healthcare professionals were randomly chosen for this study.

11. Method of approach. How were participants approached?

Individual face-to face, semi-structured interviews were performed

12. Sample size How many participants were in the study?

15 Healthcare professionals.

13. Non-participation. How many people refused to participate or dropped out? Reasons?

Setting

All the selected participants finish the study.

14. Setting of data collection.n Where was the data collected?

The interviews were in the different participants’ workplace

15. Presence of non-participants. Was anyone else present besides the participants and researchers?

No, always the interview were in presence of the participants and researchers.

16. Description of sample. What are the important characteristics of the sample?

The demographic data were published by our research group in *BMC Medicine (2013)* **11**:39

Data collection

17. Interview guide. Were questions, prompts, guides provided by the authors? Was it pilot tested?

The guide of semi-structured interview was pilot tested and published by our Group in *The Philosophy, Ethics, and Humanities in Medicine* (2013),*8:3.*

18. Repeat interviews. Were repeat interviews carried out? If yes, how many?

The interview were performed before and after CME

19. Audio/visual recording. Did the research use audio or visual recording to collect the data?

All the interview were audio recorded.

20. Field notes. Were field notes made during and/or after the interview or focus group?

Yes all the interviewers performed field notes during and after the interview.

21. Duration. What was the duration of the interviews or focus group?

On average 80 minutes.

22. Data saturation. Was data saturation discussed?

Yes, we discussed Data saturation during different steps of the research.

23. Transcripts returned. Were transcripts returned to participants for comment and/or correction?

No, the transcripts were not returned to the participants.

**Domain 3: analysis and findings**

**Data analysis**

24. Number of data coders. How many data coders coded the data?

3 coders for the hermeneutic Analysis. The coded interview were revised and discussed by the cross-functional Group (10 members) in which the coordinators are included.

25. Description of the coding tree. Did authors provide a description of the coding tree?

Yes, in figure 2 we show the codes of the research.

26. Derivation of themes. Were themes identified in advance or derived from the data?

The themes were identified in the different steps of the hermeneutic analysis and some emergent themes sprung from the interviews.

27. Software What software, if applicable, was used to manage the data?

We used Atlas.ti 6.0

28. Participant checking. Did participants provide feedback on the findings?

We present our results in several talks to the participants.

Reporting

29. Quotations presented. Were participant quotations presented to illustrate the themes / findings? Was each quotation identified? e.g. participant number

Yes, in the paper there are several quotations to illustrate the findings. The quotation are identified in our records not in the paper.

30. Data and findings consistent Was there consistency between the data presented and the findings?

Yes, we found consistency between the data and the findings.

31. Clarity of major themes Were major themes clearly presented in the findings?

Yes, the major themes were the cluster and network values and the role of consociate as amalgam of EBM-VBM

32. Clarity of minor themes. Is there a description of diverse cases or discussion of minor themes?

The minor themes as the values intuitively observed before of CME were discussed.
